# Translation as a political action: reframing ‘the deal of the century’ in the translations of the BBC

**DOI:** 10.1016/j.heliyon.2022.e08856

**Published:** 2022-01-29

**Authors:** Areej Allawzi, Hanan Al-Jabri, Deema Ammari, Sukayna Ali

**Affiliations:** University of Jordan, Jordan

**Keywords:** BBC, Discourse analysis, Narrative theory, Political action, Translation, Trump's peace plan

## Abstract

The media coverage of international events is often shaped by major actors who express their personal ideologies in the news stories they present. The ongoing conflict between Israelis and Palestinians since the mid-20^th^ century has sparked global media attention. Several studies have explored the crucial role of translation during the news coverage of this conflict. One recent development is Trump's peace plan, termed as ‘the deal of the century’, announced on 28 January 2020. It was received amidst much controversy that polarized the international community into supporters and detractors. Nevertheless, few studies have examined the media channels' representations of this plan despite the topic's sensitivity. This research article examines the media's depiction of the announcement of Trump's peace plan. It attempts to answer the question of how Trump's peace plan is narrated through different media channels to be transmitted to target audiences. Given the conflict's sensitivity, it is necessary to examine the way this plan has been represented in the media using a powerful, simultaneously flexible framework that captures the complexities of the interaction between various powers that are involved in this conflict. Mona Baker's narrative theory offers such a framework that is particularly appropriate for the current study. The analysis reveals that the BBC English source texts raise doubts about the plausibility and success of Trump's peace plan while the target text goes further by considering the peace plan as a failure since its initial proposal. Translators of the target texts used omissions, additions, lexical variations, and substitutions to frame narratives that are absent in the source texts. The Arabic text constantly describes the plan as a ‘deal’, demonstrating sympathy with Palestinians and suggesting that the proposed plan manipulates Palestinians' lives and denies them the right to decide their destiny.

## 1 Introduction

Several studies have explored the crucial role that translation plays during the media's news coverage of international conflicts (e.g. Ben-David, 2014; Bourdon, 2012; Kressel, 1987). Using Baker's narrative theory and discourse analysis, this study aims to examine the narratives generated by the British Broadcast Cooperation (BBC) to report Trump's peace plan. Baker's narrative approach has not been widely explored in the context of the Israeli-Palestinian conflict, particularly as pertaining to Trump's peace plan. Given the complexity of this conflict, it is necessary to examine the way it has been introduced using a powerful, yet, flexible framework that can capture the interaction between various powers involved in this conflict. The narrative theory offers such a framework and is particularly appropriate to use with discourse analysis to examine patterns of discrepancies between the news communicating the announcement of Trump's peace plan, also referred to as ‘the deal of the century’, and its Arabic translation by the BBC. This study aims to answer the question of how the BBC has narrated Trump's peace plan in Arabic.

The London-located BBC is one of the most prominent and established media channels. It is the oldest television and broadcasting company in the UK (Mooney and Evans, 2017, p. 170). The BBC broadcasts news in 28 different languages, including Arabic. The BBC Arabic, established in 1938, is the oldest and longest running foreign-language news services in the UK (Allawzi, 2018, p. 133).

The news agency states on its website that it produces ‘accurate and unbiased news reports, and provides information based on experience and rigorous analysis’ (BBC Arabic). This study investigates the news reports as well as the narratives generated by the translations that are produced by the BBC on Trump's peace plan using the narrative theory.

## 2 Literature review

This section gives a brief overview about Trump's peace plan and how it was perceived by Israelis and Palestinians when it was first announced. In addition, it sheds light on Mona Baker's narrative theory.

### 2.1 Trump's peace plan (the deal of the century)

On 28 January 2020, the former American President Donald Trump announced his plan to solve the on-going Israeli-Palestinian conflict, through what is known as ‘the deal of the century’, commonly known as ‘Trump's peace plan’. This plan is also formally titled ‘Peace to Prosperity: A Vision to Improve the Lives of the Palestinian and Israeli People’ (Ghanem, 2020, p. 45).

The announcement of Trump's peace plan provoked different international reactions amongst Arabs, Palestinians, and Israelis. Whereas some parties considered it a victory for Israel or even for the Israeli right wing, others interpreted it as an expression of the strength of evangelical Christians in the United States and the power of the pro-Israel lobby (Ghanem, 2020, p. 45). Some also regarded Trump's peace plan as the end of the two-state solution (Tharoor, 2020).

It did not take the Palestinian leadership long to respond publicly and officially by rejecting the proposal. The most recent polls conducted by the Palestinian Center for Policy and Survey Research indicate that 94% of the Palestinian public rejected the plan. Governments of some Arab and Western countries, the European Union and the Arab League issued statements that welcomed the efforts of Trump's administration while softly rejecting the plan, reminding the American team of the importance of the international references based on United Nations (UN) resolutions that obtained consensus (Iriqat, 2020, p. 104).

### 2.2 The narrative theory

The theoretical framework in this paper draws on Mona Baker's narrative theory introduced in *Translation and Conflict* (2006). Baker's theory is developed based on the work of Somers (1992) and Somers and Gibson (1994. p. 41) who see narrative as ‘the principle and inescapable’ mode of communication through which we experience the world not as an optional mode of communication. Hence, everything we know is the result of numerous crosscutting story-lines in which social actors locate themselves.

Baker describes translation as narration with translators as narrators (Baker, 2006, 2013, 2014; Harding, 2012) and uses the sociological aspect of the narrative approach conceiving narrative as the only means to relate to the world and our place in it (Baker, 2014, p. 159). Accordingly, Baker (2006, p. 19) defines narratives as “public and personal ‘stories’ that we subscribe to and that guide our behaviour. They are the stories we tell ourselves, not just those we explicitly tell other people, about the world(s) in which we live”. Baker's narrative approach comes from two basic assumptions. First, we have access to reality only through the modified stories we tell ourselves and others about our world. Second, the stories we narrate participate in forming reality (Baker, 2014, p. 85). Translation is, therefore, understood as ‘a form of (re-)narration that constructs rather than represents the events and characters it re-narrates in another language’ (Baker, 2014, p. 159).

Baker further highlights the importance of using the narrative theory as a framework in translation studies. The narrative theory explains that the human mind can make sense of events, whether isolated or connected, only if they are constituted as a narrative in which every element takes its place as an integral part of a ‘network’ (Baker, 2007, p. 155). Translators, likewise, locate themselves within narratives they are familiar with in their work (Baker, 2006, pp. 475–6); thus, translation becomes a construction of ‘cultural realities’ achieved by ‘the processes of narration and re-narration’ (Baker, 2013, p. 24; Dubbati and Abudayeh, 2017, p. 149).

The importance of the narrative theory is that it allows us to locate translators within the narratives they follow and that dictate their behaviour in the real world including their behaviour as translators (Baker, 2007, p. 153). Therefore, Baker's work transforms translators from being abstractions into ‘real-life individuals’ (Baker, 2007, p. 153). Furthermore, Baker's theory explains behaviour in a dynamic not static position as it considers the complexity of being embedded in opposing narratives by embedding actors within stories that change over time and space (Baker, 2007, p. 154). Another important aspect of the narrative theory is acknowledging the power of social structures and, at the same time, individual or collective resistance. It gives equal importance to stories of dominance and resistance (Baker, 2007, p. 155).

#### 2.2.1 Types of narrative

Baker (2006, p. 28; 2010, p. 350) identifies four types of social narratives: ontological, public, conceptual, and meta-narrative. Ontological or personal narratives are personal and social stories individuals relate about their position in the world, their subjective experiences and their history. This type of narrative is dependent on collective narratives that are situated in a particular social and cultural context and are transmitted to audiences through different channels such as the media.

Public narratives are shared and circulated by social institutions such as religious and educational institutions and the media. Public narratives include narratives about events whether national or international, public figures, a political group, or an ideology. These narratives circulate in any culture and can change drastically over time, given political and social changes. Translation can play a significant role in the survival of public narratives through the circulation of these narratives within diverse cultural and linguistic boundaries.

The third type of narrative is conceptual or disciplinary narratives, which are defined by social theorists, Somers and Gibson (1994, p. 62), as ‘concepts and explanations that we construct as social researchers’. Baker (2006) extends this definition to include disciplinary narratives which consist of stories and explanations that academic scholars and professionals elaborate for themselves and others about their object of inquiry.

The last kind of narrative is meta- (or master) narratives which consist of stories circulated across diverse cultures and countries. Meta-narratives can spread beyond cultural and geographical locations of any society because of the media and translators’ direct involvement (Baker, 2010, pp. 351–352). They can revolve around terrorism, Marxism, Capitalism, and Islamism.

### 2.3 Translation as activism

One of the techniques that Baker (2007) adopts from the narrative theory is the concept of reframing in order to highlight a translator's re-narration of the source text. Translators make connections which are not present in the source text and socio-politically reframe the narrative for others. These connections are established by employing several textual techniques such as lexical, semantic, and syntactic changes, and paratextual ones, such as the insertion of images and captions (Baker, 2007, p. 165). The constructed connections respond to ‘larger narratives circulating beyond the immediate text’ (Baker, 2007, p. 163). Thus, the translator, through reframing, re-narrates the story to stimulate certain reactions from target-text readers (Dubbati and Abu Abudayeh, 2017, p. 3).

Translators are not merely neutral conveyors of messages from one language into another, and they are not neutral agents occupying a liminal space between cultures and political powers. They function as ‘proxy journalists’ by highlighting certain aspects of a story and undermining others. Translation, thus, ‘does not reproduce texts but constructs cultural realities’ by interfering in the process of narration and re-narration’ (Baker, 2013, p. 24). Translators incorporate new ideas and images through translation into the target culture to promote certain narratives and challenge others (Asscher, 2021, p. 46). In this regard, Tymoczko (2009, p. 27) notes that “translation introduces discourse shifts, destabilizes received meanings, creates alternate views of reality, establishes new representations, and makes new identities possible. All these changes can produce creative results in a literary system and a culture”. Translation, thus, forms a tool in translators' hands to change the world by promoting their own accounts of political and historical events and social constructions.

## 3 Methodology

This study was conducted based on Mona Baker's narrative theory and discourse analysis to detect patterns of discrepancies between the BBC coverage of Trump's peace plan and its translation or trans-editing into Arabic. The narrative approach does not detect ‘recurrent linguistic patterns’, but it considers the body of analysis as an independent narrative representing a perspective of a certain event ‘with characters, settings, outcomes or projected outcomes, and plot’ (Baker, 2014, p. 159). A textual approach is thus employed to address the main question of how Trump's peace plan is narrated in BBC Arabic to be introduced to the target audience. Given the importance of this conflict, it becomes vital to examine the way it has been covered using a powerful and flexible framework that reflects the powers that are involved and the interaction between them. The analysis is, therefore, conducted through a comparison between the narrative constructed by BBC English, on the one hand, and its translation or trans-editing into Arabic, on the other.

The BBC is the first Europe-based network to broadcast news in the Middle East. It broadcasts news in different languages including Arabic, Persian and Urdu. BBC Arabic has its news correspondents in Arab countries and worldwide. The researchers have chosen texts from this media outlet for the following reasons. First, it has a daily coverage of international news. Second, it represents the mainstream media in the Middle East and North Africa. Finally, it has a wide range of international audiences.

The first statement that Trump made regarding the deal of the century was in English on 28 January 2020 in the White House. Thereafter, the reports were translated from English into other languages. The analysis conducted in this article is based on the 28 January 2020 announcement. The source text will be the English text, while the Arabic version will be the target text.

## 4 Results and discussion

In Table 1, the source text presents the first statement the BBC made about Trump's peace plan on 28 January 2020. The English statement comments that Trump's peace plan has been long expected without explaining the parties concerned. The source text frames the narratives that all parties that are part of the conflict, i.e. Palestinians and Israelis, are not only expecting but most likely accepting the plan.

**Table 1:** Trump releases long-awaited Middle East peace plan.

Source text: BBC English (published on 28 January 2020) Trump releases long-awaited Middle East peace plan (BBC, 2020a: Trump releases).	Target text: BBC Arabic (published on 28 January 2020) صفقة القرن: ترامب يكشف تفاصيل مبادرته في الشرق الأوسط وسط رفض فلسطيني وترحيب إسرائيلي. Trump Yakshif tafasīl mubādaratuhu fī alsharq alawsaṭ wasaṭ rafḍ fatasṭīnī wa tarḥīb isra’īlī Deal of the century: Trump reveals the details of his initiative in the Middle East amid Palestinian rejection and Israeli welcome (BBC, 2020b: Trump Yakshif; our translation).

The Arabic target text, on the other hand, uses the phrase صفقة القرن, which means ‘the deal of the century’, in the subheading. The target text also clearly states that Trump reveals the plan amid Palestinian rejection and Israeli welcome. Considering this, the English source text constructs a narrative suggesting that Trump's proposed peace plan is accepted and welcomed by the relevant parties. On the contrary, the Arabic translation reframes Trump's peace plan as controversial and disapproved by Palestinians. The narrative framed in the target text states that the plan was rejected by Palestinians and welcomed by Israelis.

The word ‘deal’ means an agreement, especially in business. Using this word in this context indicates that the plan resembles a business transaction that decides the destinies and lives of people and gambles with their lives. Choosing a word that conveys a commercial connotation and attaches it to the destinies of people indicates that Trump's peace plan bargains with Palestinians' lives and justifies their rejection.

Substituting ‘peace plan’ with ‘deal’ reflects a manipulative description and widens the scope of violations resulting from this peace plan. It can be discerned that the lexical change here is intended to reframe the narrative that this peace plan is one-sided as it only considers Israelis without considering Palestinians. Considering this, one can argue that BBC English seems to be in favour of the Israeli side, while BBC Arabic serves the interests of the Palestinian party as the Arabic narrative highlights a Palestinian reaction against Trump's peace plan.

Considering Baker's narrative theory, the target text seems to be reconstructing rather than representing the events and ‘characters it re-narrates in another language’ (Baker, 2014, p. 159). Gibson (2006) shares this view in *The Guardian*, describing the BBC's coverage of the Israeli-Palestinian conflict as ‘incomplete’ and ‘misleading’.

The source text in the following example (Table 2) communicates that the Palestinian President Mahmoud Abbas rejects the plan and describes it as a ‘conspiracy’ between the American and Israeli administrations. The word ‘conspiracy’ according to the *Cambridge Dictionary* means ‘the activity of secretly planning with other people to do something bad or illegal’ (Cambridge Dictionary). The target text, however, omits the word ‘conspiracy’ and explains that ‘the Palestinian President Mahmoud Abbas rejected the suggested plan’. The source text highlights the word ‘conspiracy’ suggesting Abbas's reaction to Trump's announcement as impulsive and hasty. Considering the other side as conspirators, the source text implies that the Palestinian side is not collaborating with or working towards finding an end to the Israeli-Palestinian conflict in order to achieve peace in the Middle East.

**Table 2:** Palestinian President Mahmoud Abbas dismissed the plan as a ‘conspiracy’.

Source text: BBC English (published on 28 January 2020) Palestinian President Mahmoud Abbas dismissed the plan as a ‘conspiracy’ (BBC, 2020c: Abbas dismissed).	Target text: BBC Arabic (published on 28 January 2020) الرئيس الفلسطيني محمود عباس رفض الخطة المقترحة قائلا: "رفضنا خطة ترامب منذ البداية ولن نقبل بدولة دون القدس". Alra’īs alfalasṭīnī maḥmūd `abbās rafaḍ alkhiṭa almuqtaraḥa qā’ilan: rafaḍna khuṭat tramb munth albidāya walan naqbal bidawla bidūn alquds. Palestinian President Mahmoud Abbas rejected the proposed plan, saying: ‘we have rejected Trump's plan since the beginning, and we will not accept a state without Jerusalem’. (BBC, 2020d: `Abbas Yarfuḍ; our translation)

On the other hand, the target text indicates that Palestinians knew of Trump's peace plan before the official announcement, but the Americans and the Israeli officials insisted on approving the plan without considering the Palestinian sentiment. This reflects that a crucial decision influencing Palestinian people's lives has been taken while denying their right of self-determination and ignoring their hopes and aspirations to have an independent state.

Highlighting the word ‘conspiracy’ as a reason to dismiss the plan, frames a narrative projecting that Palestinians do not work seriously towards achieving peace in the region. The Arabic target text, on the other hand, expresses that Palestinians will not accept any peace plan that does not recognize Jerusalem as the capital of the Palestinian state. Thus, the target text appears to generate a narrative that justifies the Palestinian's rejection of the plan proposed by Trump. Concurring, Allawzi (2018, p. 142) argues that the BBC, in contrast to its claim, does not deliver accurate information and news reports. It shapes the material and the translations of this material according to the expectations and the needs of its readers of the source and target texts. The BBC is ‘mistrusted by the public as a source of nothing but propaganda’ as it is controlled by the government (British Journalism Review, 2003, p. 5).

Table 3 below contains a title of an article by the BBC Middle East editor, Jeremy Bowen. In the title of the source text the author commences with ‘Trump's peace plan’ before stating that it is a ‘huge gamble’. According to the *Cambridge Dictionary*, the word ‘gamble’ means to do something that involves risks, with a probable loss of money or failure, in the hope of getting money or achieving success (Cambridge Dictionary). The source text does not indicate that the deal is prone to failure; rather it describes it as a ‘huge gamble’, thus, framing the narrative as though there exist chances for the success of Trump's peace plan. On the other hand, the target text starts by describing the plan as ‘the deal of the century’ which, as mentioned earlier, generates the narrative that Trump's peace plan is jeopardizing the lives of and manipulating the destinies of Palestinians. The Arabic title also states that there is a slim possibility for this plan's success فرص نجاحها ضعيفة, implying that the plan will most likely fail. Hence, whereas the source text frames the narrative that Trump's peace plan might be a success, the target text constructs a different one indicating that the plan will most likely fail, almost eliminating any potential for its success.

**Table 3:** Trump's Middle East peace plan: 'Deal of the century' is a huge gamble.

Source text: BBC English (published on 29 January 2020): by Jeremy Bowen, BBC Middle East editor, Washington. Trump's Middle East peace plan: 'Deal of the century' is a huge gamble (Bowen, 2020a).	Target text: BBC Arabic (Published on 29 January 2020) صفقة القرن: مخاطر خطة ترامب للسلام عالية وفرص نجاحها ضعيفة. Ṣafqat alqarn: makhaṭer khuṭat Trump lisalām `āliya wa furaṣ najaḥiha da`īfa. Deal of the century: The risks of Trump's peace plan are high, and the chances of its success are weak (Bowen, 2020b; our translation)

The opening sentence of the BBC English news report by Jeremy Bowen describes the atmosphere in the East Room at the White House where the press conference to make the announcement was held. It uses the word ‘party’ where attendees ‘clapped’ and ‘whooped’. The original author of the text seems to use sarcastic language mocking the atmosphere when the plan was announced. Under the image of President Trump and the Israeli Prime Minister Benjamin Netanyahu, the report explains ‘at times the atmosphere in the East Room at the White House was more a party than a news conference’ (Bowen, 2020a). Therefore, Bowen seems critical of the deal. Furthermore, Bowen's position is demonstrated in reports about breaching ‘BBC impartiality rules in the Middle East coverage’ because of bias against Israel (Foster, 2009).

As a paralinguistic tool, the BBC English version attaches a picture to the website below the headline. The picture shows the former American President, Donald Trump, speaking to the press with the Israeli Prime Minister Benjamin Netanyahu smiling while standing next to Trump. In this regard, Baker argues that paralinguistic devices such as pictures and visual resource materials can be used to frame certain narratives and deliver extra information (Baker, 2007, p. 158). The statement under the picture demonstrates the importance of Trump's peace plan to the Israeli side. Thus, the picture and the statement beneath it imply that the proposed peace plan is structured in the favour of the Americans and Israelis, without considering Palestinians.

As a translation of the opening statements, the target text uses words like أحلك ساعات الليل وفجر كاذب which mean ‘the darkest hours of nights and a lying dawn’, to describe the timing of the announcement of Trump's peace plan. Using words like ‘darkest’ and phrases like ‘lying dawn’, the translator uses a higher tone to directly criticize the announcement of the peace plan, thus, reflecting a narrative implying that the plan is a form of deceit to trick Palestinians. Furthermore, the narrative indicates that this time, after the announcement of the plan, is one of the darkest moments for Palestinians since the beginning of the Israeli-Palestinian conflict. These phrases appear under another image showing unarmed Palestinians attacked by armed Israeli soldiers. This image reframes the conflict of the Palestinian struggle against Israeli attacks. It also leads the readers of the target texts to condemn the Israeli violence and sympathize with the Palestinian resistance.

This narrative is further elaborated when the target text submits that ‘Palestinians are boycotting Trump's administration because of its professed agenda that supports Israel’. They are planning for ‘the day of anger’ or يوم الغضب. The statements provided in the target text are not delivered in the source text as the latter only conveys the stand of the Palestinian President Mahmoud Abbas. The target text makes reference to the Palestinians' rejection to deal with Trump's administration. Considering this, one can argue that although the source text only reflects the official rejection of the Palestinian administration, the target text highlights this rejection by conveying the voice of the Palestinian people and revealing their strong disapproval of the peace plan.

The source text uses words like ‘initiative’, ‘plan’, ‘deal’, and ‘document’ to refer to the plan. The word ‘initiative’ is defined as ‘a new plan or process to achieve something or solve a problem’ (Cambridge Dictionary). Using this word suggests that there were no earlier plans or projects to solve the ongoing conflict despite several attempts and a number of agreements that have been reached to solve the Israeli-Palestinian conflict since the mid-1970s (Pardo and Peters, 2010, p. 6). All these different references made to Trump's peace plan are translated as ‘deal’ صفقة in the target text which further emphasizes how Trump's suggested plan trades the lives and destinies of Palestinians to meet the interests of Israeli officials. Yet, on one occasion, the target text translates the word ‘initiative’ as مبادرة ميتة ‘a dead initiative’. Although the narrative generated by the source text questions the prospects of the peace plan, the target text goes further to regard the proposed peace plan as a failure from the beginning. Using the word ‘dead’ ميتة implies the lifeless status of Trump's plan and that there are no chances for the plan's execution.

In the same source text published by the BBC on 29 January 2020, Bowen condemns Trump's peace plan by making a reference to UN Security Council Resolution 242. Bowen submits: ‘like the UN resolution 242 that emphasizes the inadmissibility of the acquisition of territory by war, or international laws that say that occupiers cannot settle their people on occupied land’ (Bowen, 2020a, Bowen, 2020b). Nevertheless, in BBC Arabic, the translation substitutes the UN resolution with the international community by submitting بينما تعتقد معظم دول العالم أن الإسرائيليين ينتهكون القانون الدولي، which conveys that ‘while most countries of the world think that Israelis are violating the international law’. The translation is, thus, reframing the narrative that most countries are condemning Israel for violating the international law.

Given that Trump's peace plan is based on such violation, most countries will stand against the peace deal introduced by Trump. The translator is using lexical variation when occasionally substituting ‘Israeli occupation’ or ‘Jews’ with ‘Zionist occupation’ الاحتلال الصهيوني or ‘Zionism’ الصهيونية. The latter term is defined by the *Cambridge Dictionary* as ‘a political movement that had as its original aim the creation of a country for Jewish people, and that now supports the state of Israel’. Using this word delivers a political dimension of an ideology that is rejected and even resisted by most Arab and Muslim countries. In the source text, the narrative associated with Israel has national and religious implications, thus, reframing the conflict to be between Palestinians and the state of Israel. On the other hand, the target text generates a narrative that adds a political connotation and hence, reframing the conflict to be between Palestinians and Zionists inside and outside Israel. The target text indicates that the struggle is between Palestinians and the ideology of Zionism, which agitates feelings of resentment and anger not only against Israel as a state but against all countries and figures supporting Israel. Peter Beinart, an associate professor of journalism and political science at the City University of New York, best describes this in an article that rejects conflating anti-Semitism with anti-Zionism and describes it as a ‘tragic mistake’ (Beinart, 2019).

Table 4 below contains a title of an article that was published a day after Trump's peace plan was announced. The article provides a historical account on the Israeli-Palestinian conflict with all major events and turning points in this conflict. The article later describes the aim of the suggested plan as one ‘to bring peace to one of the most troubled parts in the world’ (BBC, 2020f). Providing a historical account on the conflict, without examining the plan itself and concluding the article with the aim of the peace plan to bring peace to the region, may indicate that this plan is the ideal solution for the ongoing Israeli-Palestinian conflict.

**Table 4:** Donald Trump's Middle East peace plan: The Israeli-Palestinian situation explained.

Source text: BBC English (published on 29 January 2020): Donald Trump's Middle East peace plan: The Israeli-Palestinian situation explained (BBC, 2020e situation explained).	Target text: BBC Arabic (Published on 29 January 2020) صفقة القرن: هل تقضي خطة ترامب للسلام على الرواية التاريخية لفلسطين أم أنها "حبر على ورق"؟ ṣafqat alqarn: hal taqḍī khuṭat Trump lisalām `alā ariwāya atarīkhya lifilasṭīn ‘am annaha ‘ḥibr `alā waraq’? Deal of the century: does Trump's peace plan eliminate the historical narrative of Palestine, or is it a ‘dead letter’? (BBC, 2020f: hal taqḍī khuṭat Trump; our translation)

The target text, on the other hand, does not seem to find Trump's peace plan as the ideal closure of the conflict by suggesting that the proposed peace plan might either destroy the historical narrative of Palestine, or it can only become a dead letter.

The article, thereafter, represents the reaction of some Arab columnists who criticized and rejected Trump's peace plan by describing it as ‘a big failure’ and unfair to Palestinians (BBC, 2020f: hal taqḍī). Considering this, one can propose that the target texts provide a narrative that rejects Trump's peace plan, describing it as biased, one-sided, and considerate only of the interests of the Israeli side. Baker (2010, p. 197) shares this view and explains that media channels manipulate the translation by highlighting certain parts of the story or undermining others to produce narratives that are consistent with their ideologies.

## 5 Conclusion

This study examined the BBC representation of the announcement of Trump's peace plan considering Mona Baker's (2006) narrative theory. Furthermore, it attempted to answer the question of how Trump's peace plan is narrated by different media channels to be represented to target audiences.

To respond to this, this research article used discourse analysis to reveal the patterns of discrepancies between the BBC's coverage of Trump's peace plan and its translation or trans-editing into Arabic. The narrative approach did not detect ‘recurrent linguistic patterns’, but it considered the body of analysis as an independent narrative.

The analysis above revealed that there is a consistent behaviour repeated in the Arabic target text. While the English source texts raise doubts about the plausibility of Trump's peace plan and the chance of its success, the target text goes further by deeming the peace plan as a failure since its initial proposal. Translators of the target texts used omissions, additions, lexical variations, and substitutions to frame narratives that are not produced in the source texts. The Arabic text constantly describes the plan as a ‘deal’, thereby, demonstrating sympathy with Palestinians and suggesting that the proposed plan manipulates the lives of Palestinians and denies them the right to decide their own destiny as a nation. The Arabic translation, therefore, uses certain discourse features to reject and attack Trump's peace plan.

## Declarations

### Author contribution statement

Areej Allawzi and Hanan Al-Jabri: Conceived and designed the experiments; Analyzed and interpreted the data; Wrote the paper.

Sukayna Ali and Deema Ammari: Analyzed and interpreted the data; Contributed reagents, materials, analysis tools or data.

### Funding statement

This research did not receive any specific grant from funding agencies in the public, commercial, or not-for-profit sectors.

### Data availability statement

Data included in article/supplementary material/referenced in article.

### Declaration of interests statement

The authors declare no conflict of interest.

### Additional information

No additional information is available for this paper.
